# Genome-wide discovery of InDels and validation of PCR-Based InDel markers for earliness in a RIL population and genotypes of lentil (*Lens culinaris* Medik.)

**DOI:** 10.1371/journal.pone.0302870

**Published:** 2024-05-22

**Authors:** K. M. Shivaprasad, Muraleedhar Aski, Gyan Prakash Mishra, Subodh Kumar Sinha, Soma Gupta, Dwijesh C. Mishra, Amit Kumar Singh, Akanksha Singh, Kuldeep Tripathi, Ranjeet Ranjan Kumar, Atul Kumar, Shiv Kumar, Harsh K. Dikshit

**Affiliations:** 1 Division of Genetics, Indian Agricultural Research Institute, New Delhi, India; 2 Indian Council of Forestry Research and Education-Institute of Forest Biodiversity, Hyderabad, India; 3 Indian Council of Agricultural Research-National Institute for Plant Biotechnology, New Delhi, India; 4 Indian Agricultural Statistics Research Institute, New Delhi, India; 5 Division of Genomic Resources, National Bureau of Plant Genetic Resources, New Delhi, India; 6 South Asia and China Program, International Center for Agricultural Research in the Dry Areas, National Agriculture Science Complex, New Delhi, India; 7 Germplasm Evaluation Division, National Bureau of Plant Genetic Resources, New Delhi, India; 8 Division of Biochemistry, Indian Agricultural Research Institute, New Delhi, India; 9 Division of Seed Science and Technology, Indian Agricultural Research Institute, New Delhi, India; Government College University Faisalabad, PAKISTAN

## Abstract

The systematic identification of insertion/deletion (InDel) length polymorphisms from the entire lentil genome can be used to map the quantitative trait loci (QTL) and also for the marker-assisted selection (MAS) for various linked traits. The InDels were identified by comparing the whole-genome resequencing (WGRS) data of two extreme bulks (early- and late-flowering bulk) and a parental genotype (Globe Mutant) of lentil. The bulks were made by pooling 20 extreme recombinant inbred lines (RILs) each, derived by crossing Globe Mutant (late flowering parent) with L4775 (early flowering parent). Finally, 734,716 novel InDels were identified, which is nearly one InDel per 5,096 bp of lentil genome. Furthermore, 74.94% of InDels were within the intergenic region and 99.45% displayed modifier effects. Of these, 15,732 had insertions or deletions of 20 bp or more, making them amenable to the development of PCR-based markers. An InDel marker I-SP-356.6 (chr. 3; position 356,687,623; positioned 174.5 Kb from the *LcFRI* gene) was identified as having a phenotypic variance explained (PVE) value of 47.7% for earliness when validated in a RIL population. Thus, I-SP-356.6 marker can be deployed in MAS to facilitate the transfer of the earliness trait to other elite late-maturing cultivars. Two InDel markers viz., I-SP-356.6 and I-SP-383.9 (chr. 3; linked to *LcELF3a* gene) when tested in 9 lentil genotypes differing for maturity duration, clearly distinguished three early (L4775, ILL7663, Precoz) and four late genotypes (Globe Mutant, MFX, L4602, L830). However, these InDels could not be validated in two genotypes (L4717, L4727), suggesting either absence of polymorphism and/or presence of other loci causing earliness. The identified InDel markers can act as valuable tools for MAS for the development of early maturing lentil varieties.

## Introduction

Lentil (*Lens culinaris* Medik.) is an important cool-season legume crop of rainfed agriculture [1] and globally its cultivation spans approximately 5.01 million hectares (Mha), yielding 6.54 million metric tons (Mt) with a productivity of 1305 kg/ha. In India, lentil is cultivated in nearly 1.35 Mha area, yielding 1.18 Mt of production with 871.5 kg/ha productivity [2]. Lentil plays a distinctive role within cereal-based cropping systems, contributing significantly to the enhancement of human, animal, and soil health. Lentil contains very low levels of antinutritional factors, thus making it an excellent source of protein and amino acids that complement the cereal protein [3].

Lentil crop remains susceptible to drought and heat stress, particularly during grain filling stage, which results in forced maturity and reduced yield. With climate change projections indicating increased frequency and intensity of drought and heat stress [4], there is an urgent need to develop early-maturing lentil cultivars capable of surviving under such challenges with minimum or no yield penalty [5]. To thrive in cereal-based cropping systems, especially in rice fallow areas in South Asia, there is a demand for extra-early varieties that mature in less than 100 days.

Classical breeding methods have achieved success in mainstreaming some monogenic traits in lentils that are relatively easy to manage. However, quantitative traits are often influenced by environmental conditions, the conventional approach proves less precise [6]. This necessitates the deployment of recent genomics tools including development and deployment of linked markers for various traits of interest. The identification of molecular markers associated with QTLs has not only shed light on the genetic underpinnings of complex traits but has also endeavoured to enhance the precision and efficiency of crop improvement through MAS [7,8].

The complexity of the lentil genome, comprising n = x = 7 chromosomes, with nearly 4.2 Gb genome size, presents a real challenge to the breeders for the identification of linked markers and QTLs with the trait of interest. Consequently, it becomes essential to develop robust molecular markers suitable for genetic mapping and MAS in lentils. Recent advancements in sequencing technologies have introduced high-density SNP markers to the lentil research community [9–11].

The emergence of whole genome resequencing (WGRS) has paved the way for the generation of copious amounts of sequence data at a relatively economical cost, thereby unlocking genetic markers that were hitherto inaccessible. Single-nucleotide polymorphisms (SNPs) and Indels have emerged as the most prevalent types of genetic markers identified through WGRS [12,13]. InDel markers with moderate size differences can be efficiently used for PCR-based primer designing, amplification, and visualization using agarose gel electrophoresis. These markers have been used for various genetic studies in various crops including soybean [13], and chickpea [14]. However, in lentils, the InDels are very scantily reported in the public databases [15–17] and no PCR-based InDel markers have been developed till now. This study used QTL-seq to map QTLs and identify the candidate genes associated with earliness and identify and validate the PCR-based InDel markers linked with earliness trait in lentils.

## Materials and methods

### Plant materials and Phenotyping

A total of 230 RILs (F_4:5_) were developed through single seed descent (SSD) method from a cross between the Globe Mutant [late maturing, days to maturity (DTM) = 135–145 d] as the female parent and L4775 (early maturing, DTM = 95–105 d) as a male parent. These RILs, along with nine lentil genotypes significantly varying for maturity duration were used. The five early (L4775, ILL7663, Precoz, L4717, L4727) and four late genotypes (Globe Mutant, MFX, L4602, L830) along with the RILs were planted during the *rabi* 2022–23 in the research farm of ICAR-Indian Agricultural Research Institute, New Delhi, India, which is situated at a latitude, longitude and altitude of 28.080°N, 77.120°E, and 228.61 meters AMSL, respectively. The observations were recorded for the days to flowering (DTF) (S1 Fig, S1 Table).

### InDel calling

WGRS data was used for InDel calling which was obtained from the QTL-seq study for earliness involving Globe Mutant, early bulk (DTM mean: 99.0 d, DTM range: 96–100 d), and late bulk raw reads (DTM mean: 139.9 d, DTM range: 136–144 d) [NCBI sequence read archive (SRA) number PRJNA915231; https://www.ncbi.nlm.nih.gov/bioproject/PRJNA915231]. The paired-end (PE) reads, consisting of 2×151bp PE sequences and an average sequencing depth of 12.9× were used. The clean reads were aligned to the lentil reference genome CDC Redberry Genome Assembly v2.0. The InDels variations were subsequently identified for the Globe Mutant, late bulk, and early bulk at each base position using the bcftools mpileup and bcftools call [18]. The resulting VCF file, encompassing InDels information was then employed for subsequent analyses.

### InDel Annotations

The SnpEff binary database file was generated using the lentil genome annotation file (gff3) and the reference genome. This database was used to annotate the effects of InDels by region effect (high, moderate, low, and modifier), and are classified into downstream, exon, intergenic, intron, splice site acceptor, splice site donor, splice site region, upstream, 3’UTR, and 5’ UTR. The localization of InDel was based on the annotation of the gene models of the CDC Redberry Genome Assembly v2.0 [19] reference genome [20].

### Filtering of InDels and development of primers for selected InDels

During InDel analysis, the VCF file containing InDel size variations <20 bp were systematically filtered out, while InDels ≥20 bp were designated as potential candidates for subsequent evaluation via agarose gel electrophoresis. All the indels were then identified located on all the seven lentil chromosomes (S2 Table). The sequences flanking the InDel, comprising 400 bp upstream and downstream, were extracted from the reference sequences of the CDC Redberry Genome Assembly v2.0, and PCR-based primers were designed using the NCBI Primer-BLAST tool (https://www.ncbi.nlm.nih.gov/tools/primer-blast/). The primer designing criteria include: primer size (18–25 bp), GC content (40–60%), Tm difference between forward and reverse primers (up to 3°C), and PCR products (100–500 bp).

### DNA extraction and PCR

Young lentil leaves were collected from RILs and the nine selected genotypes 40–45 days after sowing and DNA was isolated using CTAB method [21]. Approximately 100 mg leaf was grounded in 4.0 mL DNA extraction buffer consisting of 5% CTAB, 1M Tris–HCl (pH 8.0), 5M NaCl, 0.5M EDTA, 0.2% β-mercaptoethanol, and 20 mM RNaseA. The homogenate (0.5 mL) was transferred to a 1.5 mL microfuge tube and incubated (60°C; 60 min) in a water bath. Subsequently, 0.6 mL chloroform: isoamyl alcohol (24:1 v/v) was added, vortexed (30 sec), and centrifuged (MIKRO 200 R centrifuge, Hettich, North America) (10,000 RPM, 24°C, 10 min). The aqueous phase was transferred to a 1.5 mL microfuge tube and precipitated with 0.6 mL isopropanol and kept overnight at -20°C. This was then centrifuged (10,000 RPM at 4.0°C for 5.0 min) and DNA pellet was washed with 70% ethanol (0.6 mL), air-dried, and re-suspended in TE buffer (0.2 mL).

For PCR amplification, 80 ng genomic DNA was used for 10 μL reaction volume containing 2×Taq Buffer A, 1.0 mM dNTP mix, 0.15 μL Taq polymerase (Meridian Bioscience), and 2.5 pmoles of forward and reverse primers. PCR conditions included initial denaturation at 94°C for 3 minutes, followed by 35 cycles of 94°C for 30 seconds, 55–60°C for 30 seconds, and 72°C for 60 seconds, with a final extension step at 72°C for 8 minutes. The resulting PCR products were separated on a 3.5% agarose gel, stained with ethidium bromide in 1X TBE buffer, using 150V for 2.5h electrophoresis. The gels were visualized and photographed using an AlphaImager MINI equipped with an Ultraviolet transmission analyzer.

### InDel marker for earliness trait

The InDel marker named, I-SP-356.6 was used for the genotyping of 230 RILs and 09 lentil genotypes differing for maturity duration. Single marker analysis was performed using simple linear regression analysis for I-SP-356.6 InDel marker’s data. The InDel marker genotypic data served as an independent variable, while DTF data for *rabi* 2022–2023 as the dependent variable. In addition, the InDel markers I-SP-356.6 (linked to *LcFRI* gene) and I-SP-383.9 (linked to *LcELF3a* gene) were also used for the validation on 09 lentil genotypes differing for DTF [22].

### Digital gene expression analysis

For *in silico* validation, digital gene expression analysis was performed for the *LcELF3a* and *LcFRI* genes using *Arabidopsis* orthologs. The gene expression pattern has been searched using ’Expression Angler’, a new BAR tool for mining expression data online for these genes [23].

## Results

### Identification of InDels between Globe Mutant and L4775

**Fig 1:**
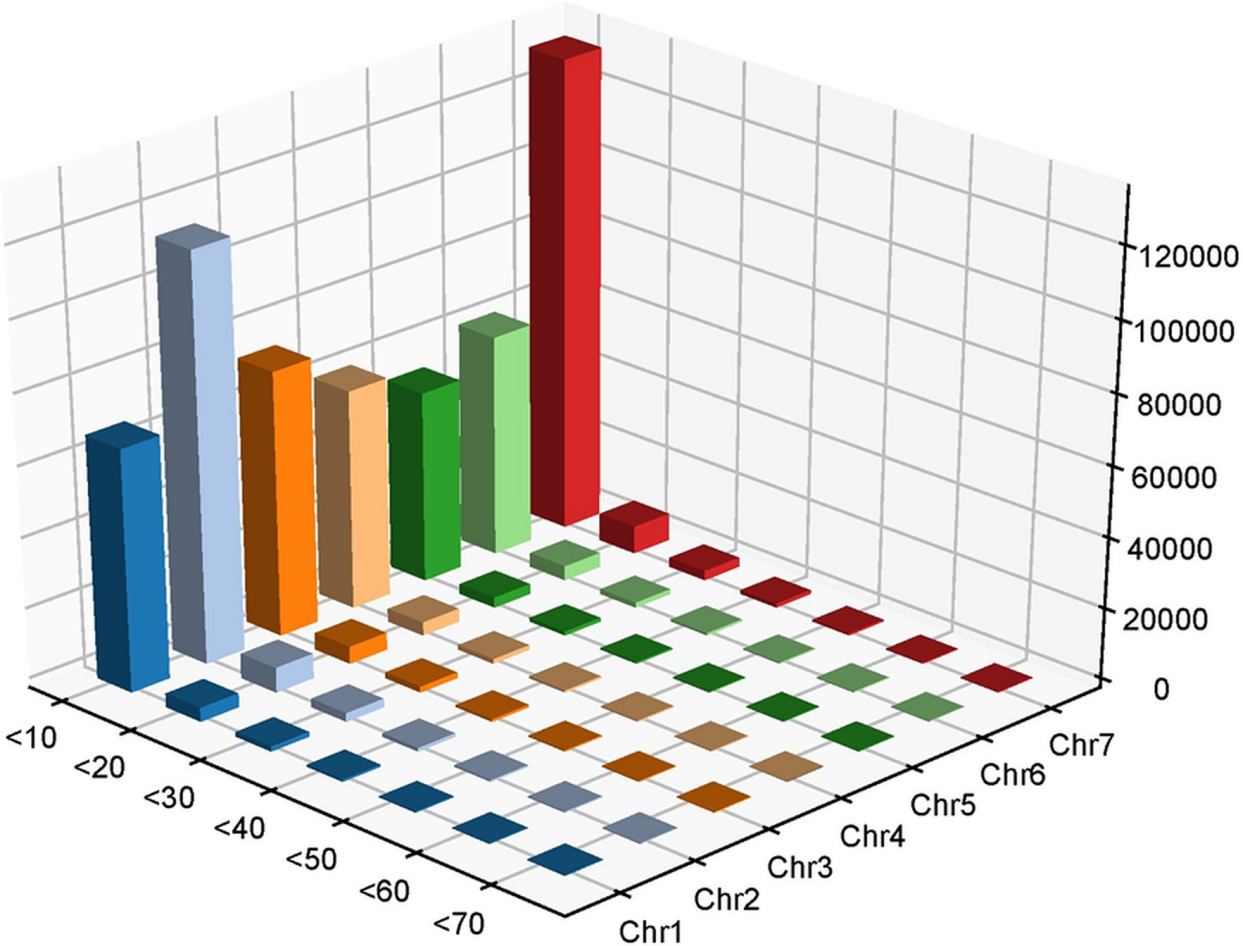
Distribution of InDels between the lentil genotypes Globe Mutant and L4775 on the seven lentil chromosomes.

**Fig 2:**
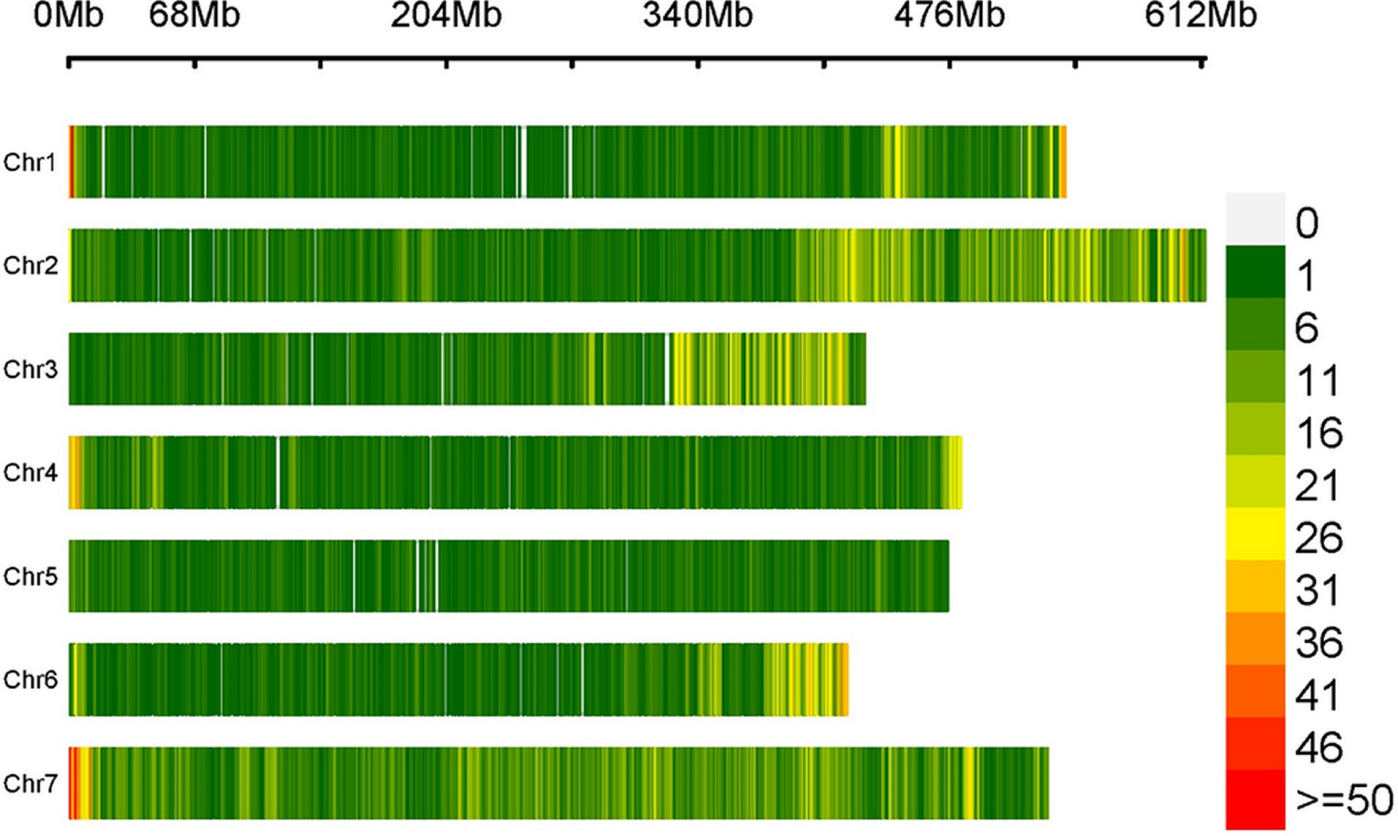
InDels density plot showing several InDels within 1Mb window size distributed on the seven lentil chromosomes.

The high-quality clean reads were aligned to the CDC Redberry Genome Assembly v2.0, covering 99.9% of the reference sequence with a mean sequencing depth of 12.9×. A total of 734,716 InDels were identified between the EB and LB, attributed to the genomic variations between the parental genotypes (Globe Mutant and L4775). Of these, 376,078 were deletions, while 358,638 were insertions and 42,298 were located on unanchored sequence Unitigs/scaffolds. The number of InDels on each chromosome ranged from 66,992 (Lcu.2RBY.Chr5, 475.0 Mb) to 157,954 (Lcu.2RBY.Chr7, 529.1 Mb), with a genome-wide average density of ~1 InDel per 5,096 bp (Table 1). The details of insertions and deletions across the 07 lentil chromosomes are illustrated in Figs 1 and S2, and Table 2. The InDel distribution pattern suggests their uneven distribution throughout the genome (Fig 2).

**Table 1:** The distribution and number of InDel polymorphisms between the lentil genotypes Globe Mutant and L4775.

Chromosome	Length	Variants	Variants rate
Lcu.2RBY.Chr1	53,83,62,633	84,123	6,399
Lcu.2RBY.Chr2	61,40,89,212	1,38,915	4,420
Lcu.2RBY.Chr3	43,01,15,100	90,562	4,749
Lcu.2RBY.Chr4	48,22,16,434	77,444	6,226
Lcu.2RBY.Chr5	47,50,07,465	66,992	7,090
Lcu.2RBY.Chr6	42,05,84,759	76,428	5,503
Lcu.2RBY.Chr7	52,90,90,306	1,57,954	3,349
Unitigs	25,47,57,864	42,298	6022
**Total**	**3,74,42,23,773**	**7,34,716**	**5,096**

**Table 2:** Distribution of InDels between Globe Mutant and L4775 along the seven lentil chromosomes as per their size variations.

Chromosomes	InDels size (bp)							
	≤10	≤20	≤30	≤40	≤50	≤60	≤70	≤80
Chr1	68576	3770	1181	399	119	42	1	0
Chr2	115286	6835	2246	781	259	80	10	0
Chr3	74915	4659	1457	496	161	35	6	0
Chr4	62048	3526	1100	346	127	45	0	0
Chr5	54033	2696	874	277	97	26	2	0
Chr6	62551	3846	1166	422	123	37	3	0
Chr7	132906	7574	2467	892	341	97	16	1
**Total**	**570315**	**32906**	**10491**	**3613**	**1227**	**362**	**38**	**1**

### Annotation and classification of identified InDels

The InDels were categorized as low impact (0.097%), modifier (99.454%), moderate (0.158%), and high impact variation (0.29%). InDels were more abundantly observed in the intergenic region (74.937%) followed by upstream (9.812%) and downstream (9.602%) regulatory regions (URR and DRR), intron region (3.851%), exon region (0.429%), 3’UTR (0.755%), 5’ UTR (0.49%), splice site region (0.097%), splice site acceptor region (0.008%), and splice site donor region (0.012%) (S3 Fig, Table 3). Collectively, 2,652 high-impact InDels were identified, of which 52 cause gain of stop codon, 71 cause loss of stop codon, 57 cause loss of start codon, while 2431 cause frameshift variations. A total of 3,919 InDels cause deletion/insertion of amino acids, while 69, 106, and 888 InDels affect essential splice donor sites, splice acceptor sites, and splice site regions, respectively. Modifier InDels were mostly present in the upstream and downstream gene variants, while the low-impact InDels were present in the intron and splice site regions.

**Table 3:** Genome-wide number of InDels by region.

Type	Percent
Downregulated (DRR)	9.60%
EXON	0.43%
INTERGENIC	74.94%
INTRON	3.85%
SPLICE_SITE_ACCEPTOR	0.01%
SPLICE_SITE_DONOR	0.01%
SPLICE_SITE_REGION	0.10%
Upregulated (URR)	9.81%
3’UTR	0.76%
5’UTR	0.49%

### Development and validation of InDel marker for earliness in lentil

**Fig 3:**
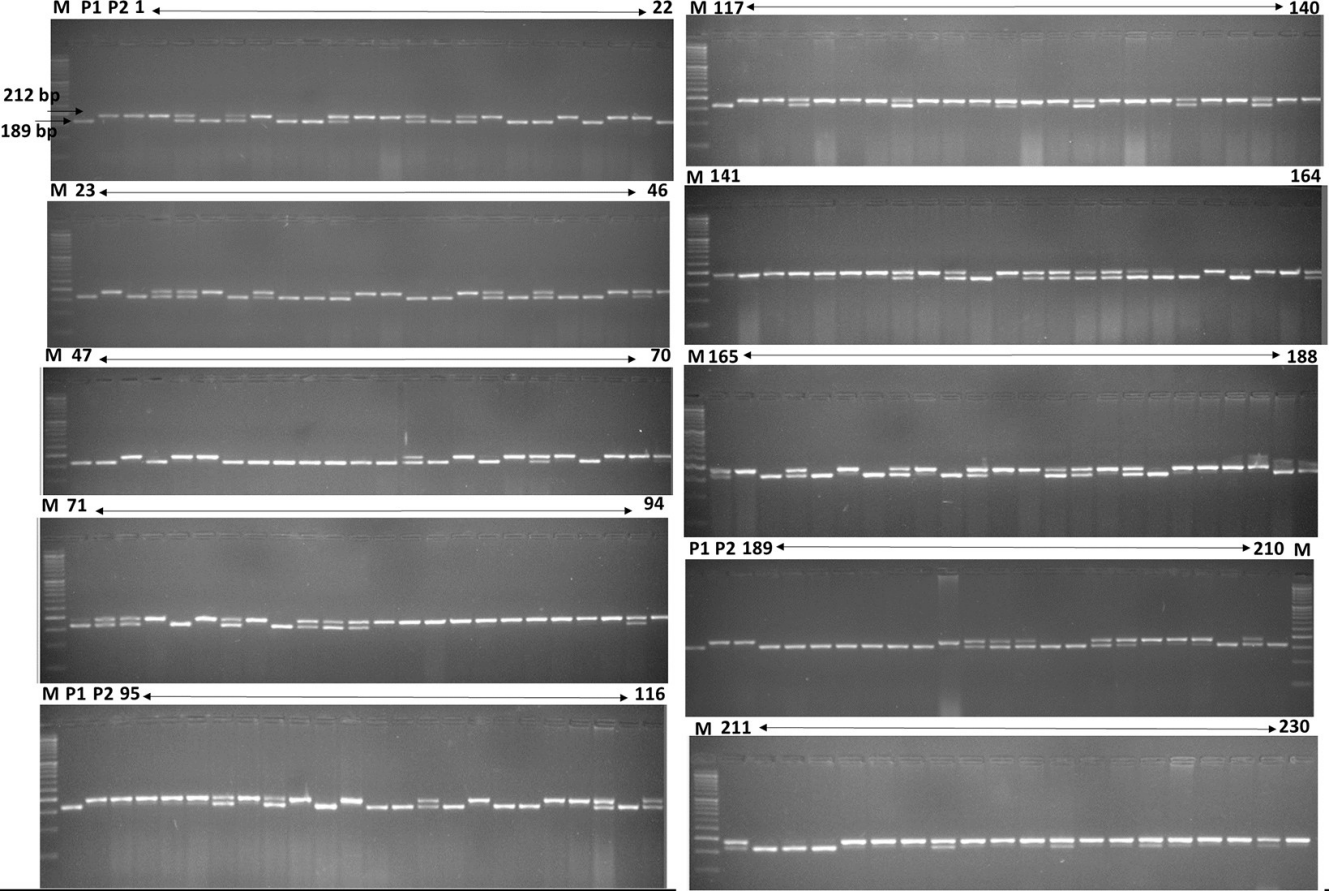
Genotyping of 230 RIL individuals using InDel marker I-SP-356.6.

To evaluate the usability of the InDel marker for genetic mapping, a PCR-based InDel marker named, I-SP-356.6 was developed based on its proximity (174.5Kb) to the *LcFRI* gene (position 356687623) on chr. 3. This marker exhibited 23-bp deletion exclusive to Globe Mutant (Table 4) (NCBI BioProject number PRJNA915231). The flanking sequence (400 bp upstream and downstream) was retrieved from the reference sequences of the CDC Redberry Genome Assembly v2.0 and PCR primers were designed (Table 5). This marker amplified a 189 bp band in late parent (Globe Mutant) and a 212 bp band in the early parent (L4775). Upon testing within the RIL population, it showed perfect co-segregation with the flowering trait (Fig 3). The single marker analysis using simple linear regression with two classes (EE = 1 and Ee/ee = 0) displayed a very high 47.7% PVE for this InDel marker. Here, marker data was used as an independent variable, and DTF as the dependent variable.

**Table 4:** InDel used for marker development.

InDel position	Globe Mutant sequence	Length (b)	L4775 sequence	Length (b)	InDel size difference
356687623	TAGTTAACTA	10	TAG TTA ACT ATA TTA GTT AAC TAC AGT TAA CTA	33	23

**Table 5:** InDel primers sequence details.

Primer name	Sequence
I-SP-356.6-F	GGA GGA TCT TCC TTT CTT CTT GTG
I-SP-356.6-R	GCT GCT TTT CAA CCA CCA TCC

### Validation of InDel polymorphisms associated with earliness in lentil genotypes

**Fig 4:**
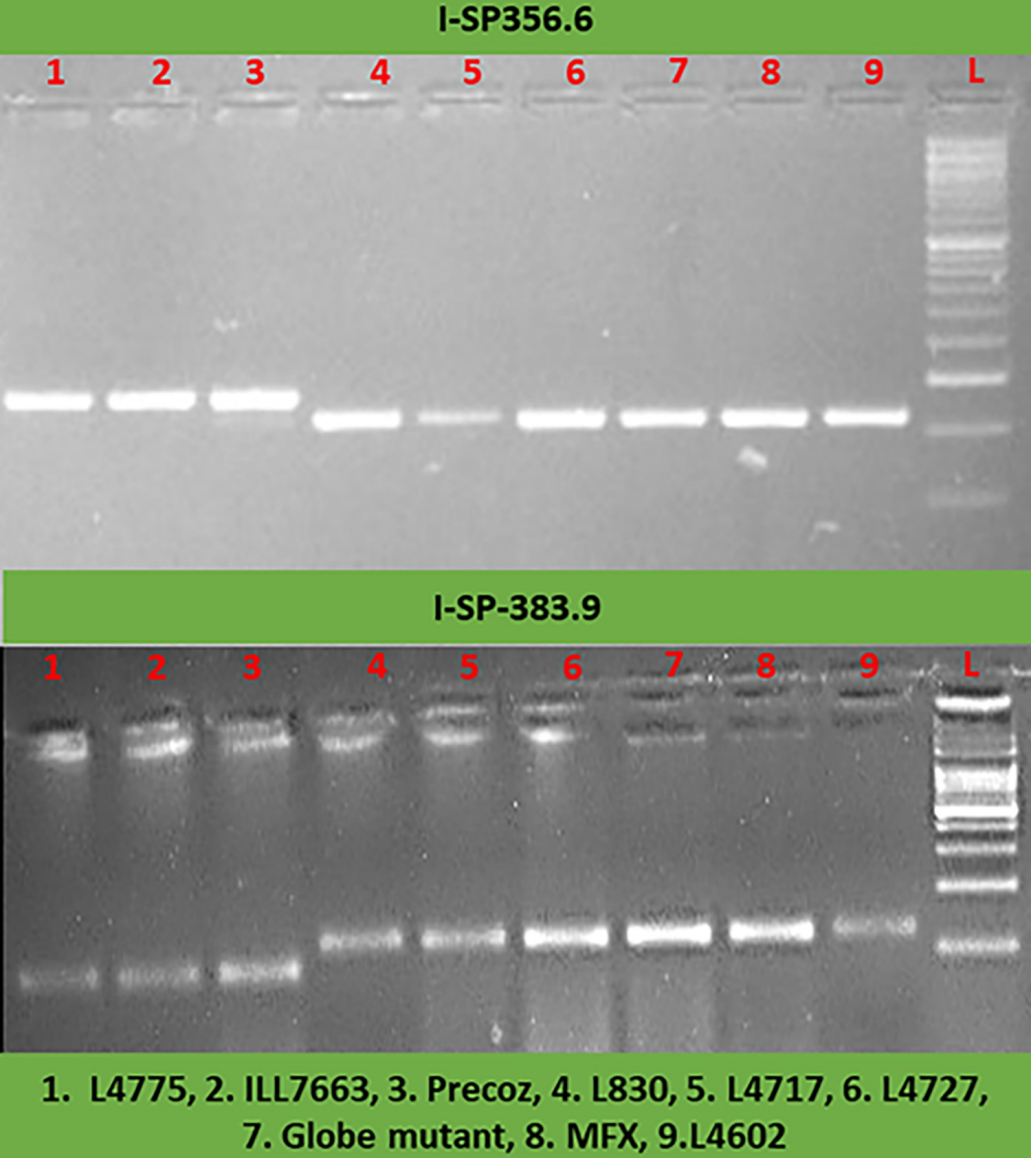
InDel marker polymorphism details in nine lentil genotypes differing for maturity duration.

The InDel marker I-SP-356.6 linked with earliness was validated using nine lentil genotypes differing for maturity duration as late maturing (Globe Mutant, MFX, L4602, L830) and early maturing (L4775, ILL7663, Precoz, L4717, L4727) (Fig 4). The marker was successfully validated for earliness among seven genotypes, consisting of three early (L4775, ILL7663, Precoz) and four late genotypes (Globe Mutant, MFX, L830 and L4602). However, this marker could not be validated in two genotypes (L4717 and L4727). Similar results were also observed for another InDel marker I-SP-383.9 which was located near *LcElf3a* gene (chr. 3) when tested on these nine genotypes.

### Validation of identified *LcELF3a* and *LcFRI* through digital gene expression analysis

**Fig 5:**
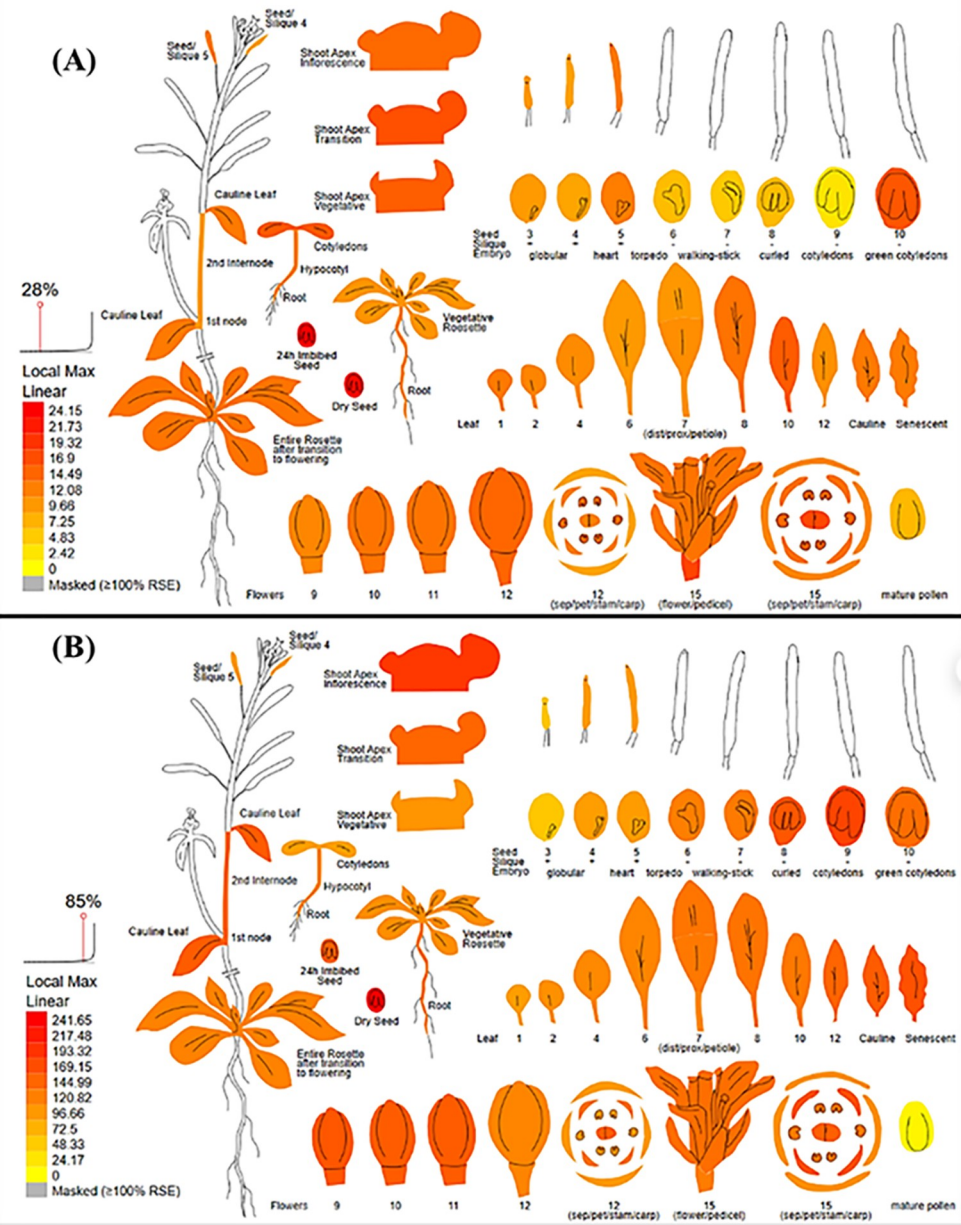
Digital gene expression analysis of (A) *FRI*, and (B) *ELF3* genes using *Arabidopsis* orthologs.

Digital gene expression analysis is used for the in-silico validation of identified candidate genes using *Arabidopsis* orthologous (compared with the *Lens culinaris* Medik. genome) by identifying which part of the plant gets affected by the candidate genes. This analysis clearly showed very high expression of *FRI* (AT4G00650) and *ELF3* (AT2G25930) genes, in leaves, flower buds, shoot apex, and flower tissues (Fig 5).

## Discussion

In the context of climate change, it is crucial to develop lentil varieties exhibiting increased productivity and ability to withstand environmental pressures, both abiotic and biotic, including extra-early maturity. Achieving this requires the use of modern genomic tools and marker-assisted selection (MAS). Despite these advancements, PCR-based InDel markers have not been developed for lentils. This study for the first time reports the development and validation of InDel markers linked with earliness trait in lentils.

### Identification of InDels between late flowering (Globe Mutant) and early-flowering (L4775) lentil genotypes

A thorough examination to detect the insertions and deletions between early-flowering bulk and late-flowering bulk, using CDC Redberry Genome Assembly v2.0, yielded valuable insights. Approximately 99.9% of high-quality reads (HQR) aligned with the lentil reference genome, a crucial factor for accurate variant calling. The mean sequencing depth (12.9x) further ensured the data’s robustness and reliability. In comparison, Indel-seq in pigeonpea (Fusarium wilt and sterility mosaic disease; [24] and QTL-seq in bitter gourd (gynoecious sex expression; [25] demonstrated 84.9% and 98% of HQR aligned with the average depth of 11.8x and 56.9x, respectively.

A total of 734,716 InDels were identified, indicating substantial genetic differences between the two lentil genotypes (Globe Mutant and L4775). The presence of such a large number of InDels hold potential for identifying linked markers for various traits of interest in lentils, particularly when studied across diverse mapping populations. Similarly, Indel-seq in pigeonpea revealed 211,603 InDels between high trait bulk (HTB) and low trait bulk (LTB) [24], while QTL-seq in cucumber identified 18,646 InDels between resistant-pool and susceptible-pool [26]. Notably, 42,298 detected InDels were located on unanchored sequence Unitigs/scaffolds, representing less-characterized regions of the genome. This underscores the challenges in gaining a comprehensive understanding of the entire lentil genome as also identified in pigeonpea, where 122,342 InDels were found on unanchored sequence scaffolds [24]. Examining the distribution of InDels across lentil chromosomes revealed that Lcu.2RBY.Chr7 has highest number of InDels (157,954), while Lcu.2RBY.Chr5 had the lowest (66,992). This uneven distribution implies that certain genomic regions may be more prone to genetic variation, potentially influenced by selective pressures or other factors, as well as variations in chromosome length. Similarly, in soybean, the identified InDels on each chromosome ranged from 1466 (Gm11, 34.7 Mb) to 3397 (Gm15, 51.7 Mb) [13]. The genome-wide average InDel density was nearly one InDel per 5,096 bp (or 196.2 InDels per Mb), while in soybean, this was only 52.4 InDels per Mb [13]. This indicates that the lentil genome exhibits greater genetic variation, providing an opportunity to compare the genetic diversity in other lentil genotypes or related *Lens* species.

### Annotation and classification of identified InDels in lentil

In lentils, the presence of InDels was notably abundant in intergenic regions, a pattern also observed in pear [27] and papaya [28]. This suggests that non-coding regions demonstrate a higher capacity to accommodate such structural variations. Furthermore, InDels were identified in regulatory regions, both upstream and downstream of genes, albeit at a lower frequency. This distribution of InDels indicates their regulatory roles in influencing the expression of lentil genes. The grouping of identified InDels into four impact categories offered insights into the functional consequences of these genetic variations. The majority of InDels fell into the modifier category, suggesting that they may exert subtle effects on gene function [20]. Similar observation is noted in rice, where 94.1% of variants belong to the modifier category [29]. Conversely, high-impact InDels are relatively infrequent but they carry significant implications for gene function. The study identified 52 InDels causing a gain of stop codons, while 71 led to a loss of stop codons in gene transcripts. Such alterations may disrupt the normal termination of protein synthesis, thereby affecting the function of resulting protein. Furthermore, 57 high-impact InDels resulted in the loss of start codons, potentially impeding the initiation of respective protein synthesis. Additionally, 2,431 high-impact InDels exhibited frameshift variations, which have the potential to substantially alter the amino acid sequence of a protein. This alteration could likely lead to the synthesis of non-functional proteins. Similar trend was observed in rice, where frameshift mutation variants accounted for 2518 of the total 3524 high-impact group variants [29].

The influence of InDels on essential splice donors, acceptors, and splice sites appears to impact mRNA splicing, leading to alternative splicing patterns and thereby generating diverse protein isoforms. These protein isoforms may have various implications at the cellular function level [20,30]. Modifier InDels are predominantly variants located either upstream or downstream of genes, playing a role in regulating gene expression. This regulation may occur by influencing the binding of transcription factors or other regulatory elements. The presence of these InDels in such regions underscores the importance of considering not only coding sequences but also regulatory regions when assessing the functional impact of genetic variations. Low-impact InDels are primarily situated in intronic and splice site regions. Individually, they may exert minimal effects on gene function, but their cumulative impact on splicing patterns and regulatory processes should not be overlooked. Understanding their role in gene regulation may offer insights into subtle yet potentially important genetic mechanisms [20,31,32].

### Development of InDel marker for earliness trait in lentil

The InDel marker, I-SP-356.6, was developed from the QTL-seq data by pinpointing the target region that regulates earliness in lentils, utilizing the information available on NCBI (SRA BioProject number PRJNA915231). The I-SP-356.6 marker is closely linked with the *LcFRI* gene, a known regulator of earliness located on chromosome 3. Through PCR-based amplification, the early and late genotypes were clearly differentiated, producing a 189 bp band in the late-flowering parent (Globe Mutant) and a 212 bp product in the early-flowering parent (L4775).

The I-SP-356.6 marker showed a substantial PVE (47.7%) for the flowering trait in the RIL population, as revealed through single marker analysis. Similarly, in pumpkin, the marker R2_51047 exhibited a PVE of 29% within the mapping region of *qfffn9-a*, linked to the first female flowering node (FFFN) [33]. In rice, an InDel marker, ZMEH_1, explained 86.0% PVE for heading date [34]. Using simple PCR technology, the high PVE for I-SP-356.6 marker makes it a promising marker for distinguishing between early-flowering genotypes with a non-functional *LcELF3a* allele and late-flowering lentil genotypes. The co-segregation of the marker with the flowering phenotype suggests a close linkage between the marker and the region governing flowering time in lentils (Fig 3).

Thus, the InDel marker I-SP-356.6 emerges as a valuable tool for the identification and selection of early maturing lentil genotypes, a crucial factor for escaping terminal heat stress, thereby enhancing yield, and bolstering resilience to diverse environmental conditions. Similarly, in rice, an InDel marker, ZMEH_1, was developed for early heading [34]. This marker holds potential for integration into marker-assisted breeding programs focused on developing early-maturing lentil genotypes, particularly well-suited for rice fallow conditions. Furthermore, these findings lay a foundation for subsequent studies delving into the molecular mechanisms governing earliness traits. Notably, in *A*. *thaliana*, the allelic variation of *FRI* contributes around 70% to the variations in flowering time [35].

### Validation of InDel polymorphisms associated with earliness in lentil accessions

The successful PCR amplification of InDel markers I-SP-356.6 and I-SP-383.9 across nine lentil accessions provides experimental confirmation of their linkage with earliness traits in lentils. This ultimately affirms the reliability of these InDel markers for trait mapping. In cabbage, a diagnostic InDel marker for head-splitting resistance was developed [36]. Similarly, in soybean, the *CRINKLY LEAF* locus was mapped on chromosome 7 (360 kb) using InDel markers [13]. In ornamental kale, the purple leaf gene (*BoPr*) was fine-mapped using InDel markers [37]. Thus, the identified InDels have the potential to map various traits based on the candidate gene information available for the traits located on different chromosomes. The I-SP-356.6 marker clearly distinguished the flowering duration trait in 07 lentil genotypes including 03 early (L4775, ILL7663, Precoz) and 04 late genotypes (Globe Mutant, MFX, L4602, L830). This suggests its applicability for MAS in lentils for earliness. Furthermore, its proximity to the *LcFRI* gene suggests its potential role in regulating flowering time, opening avenues for further detailed functional characterization of this gene. The *FRI* gene, known for regulating flowering time, has been reported in *Brassica rapa* [38], *Arabidopsis* [35,39], and *Lupinus albus* for early flowering [40]. However, the validation of markers (I-SP-356.6 and I-SP-383.9) could not be achieved in two genotypes (L4717, L4727), indicating presence of other gene(s) regulating earliness. Thus, these genotypes may serve as a potential sources harboring additional loci controlling early maturity. Similarly in *Brassica napus*, six genotypes out of 256 accessions could not be validated for earliness [41], highlighting the involvement of other loci controlling flowering in those genotypes. The validated InDel markers (I-SP-356.6 and I-SP-383.9) can be a valuable tool for selecting genotypes with loci governing early maturity in lentils. Furthermore, there is a need to identify other loci governing earliness and subsequently develop extra-early maturing genotypes by pyramiding such loci in lentils through MAS. In rice, lines with two or three pyramided genes exhibited a wider resistance spectrum than monogenic lines for blast resistance [42]. Digital gene expression analysis also revealed elevated expression of *FRI* and *ELF3* genes, particularly in leaves, shoot apex, and flower buds, thereby regulating flowering time in lentils.

## Conclusions

The in-depth examination of lentil genome revealed 734,716 InDels between the Globe Mutant and L4775, highlighting the presence of substantial genetic variations. A specific InDel marker, I-SP-356.6, tightly linked with the early maturity was identified. This marker exhibited distinct amplification pattern between early and late genotypes, as well as in the RIL population. The perfect co-segregation of I-SP-356.6 with earliness in an RIL population reaffirms its efficiency as a genetic marker. The I-SP-356.6 can be used as a valuable tool for selecting early maturing genotypes through MAS using simple PCR technology. The findings also indicated the presence of additional loci governing earliness in other lentil genotypes.

## Supporting information

S1 Fig Frequency distribution of 230 RIL individuals for days to flowering. (TIF)

S2 Fig Genome-wide distribution of InDels identified between Globe Mutant and L4775 across seven lentil chromosomes through variants histogram. (TIF)

S3 Fig Genome-wide localization of several InDels based on the genomic regions. (TIF)

S1 Table Days to flowering (DTF) of RIL population and selected genotypes. (DOCX)

S2 Table Chromosome based InDel primers details in lentil. (DOCX)

S1 Raw images (PDF)
