# Supplementary figures and images for: Genome-wide discovery of InDels and validation of PCR-Based InDel markers for earliness in a RIL population and genotypes of lentil (Lens culinaris Medik.)
Source: PLoS One. 2024 May 22;19(5):e0302870. doi: 10.1371/journal.pone.0302870 (PMC11111061; doi:10.1371/journal.pone.0302870)

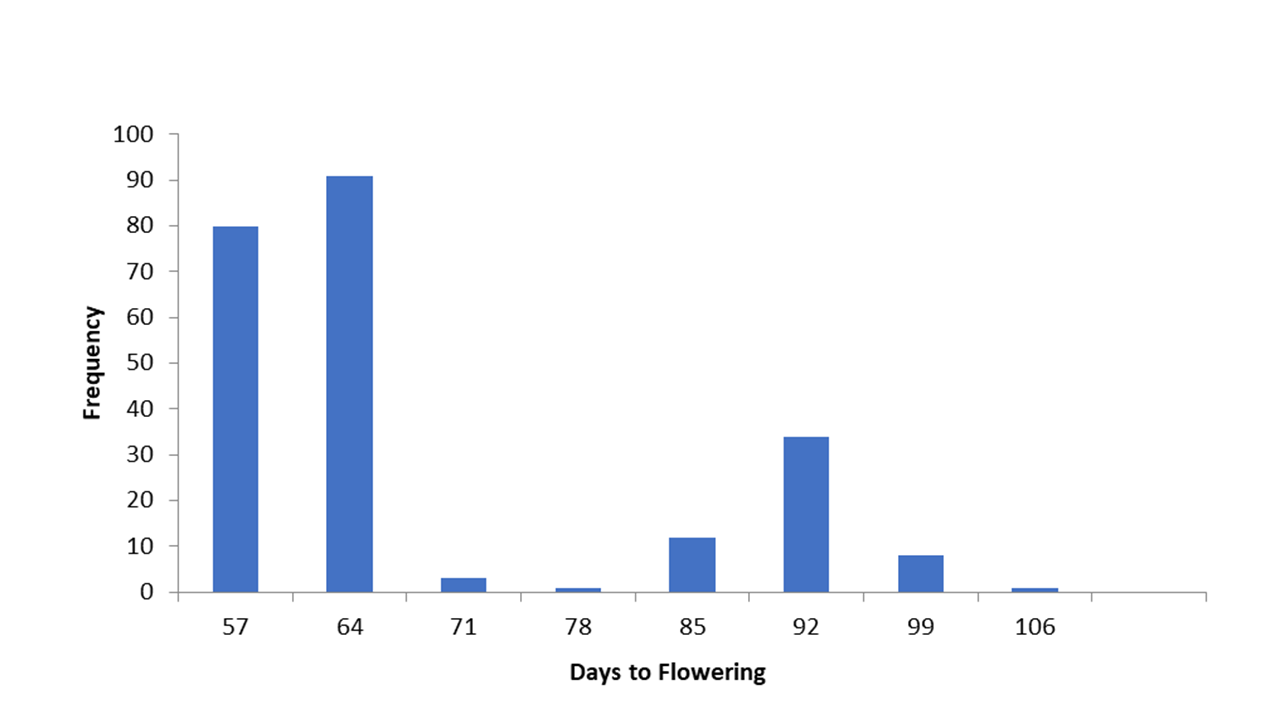

Supplement: S1 Fig — (TIF) [file pone.0302870.s001.tif]

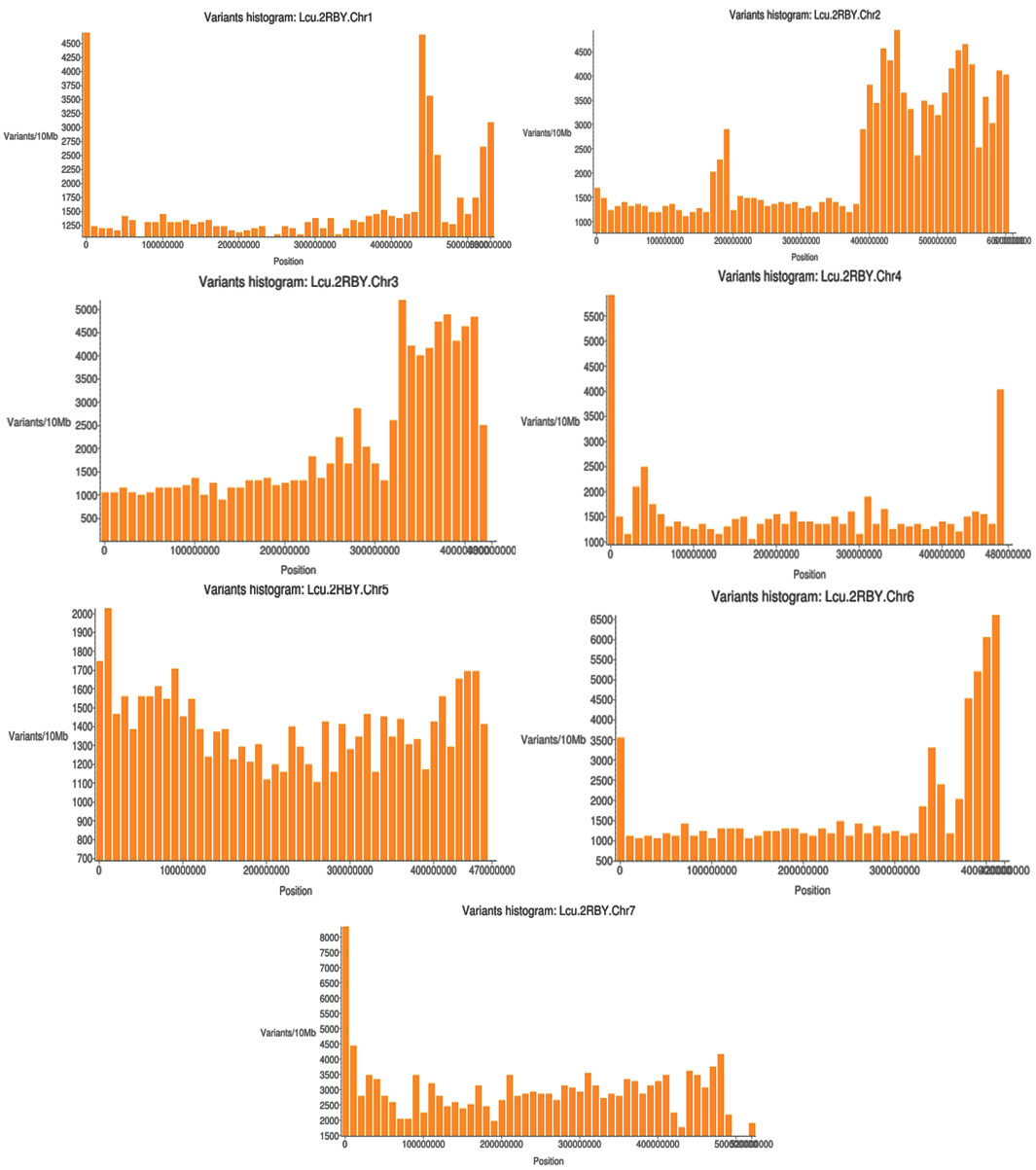

Supplement: S2 Fig — (TIF) [file pone.0302870.s002.tif]

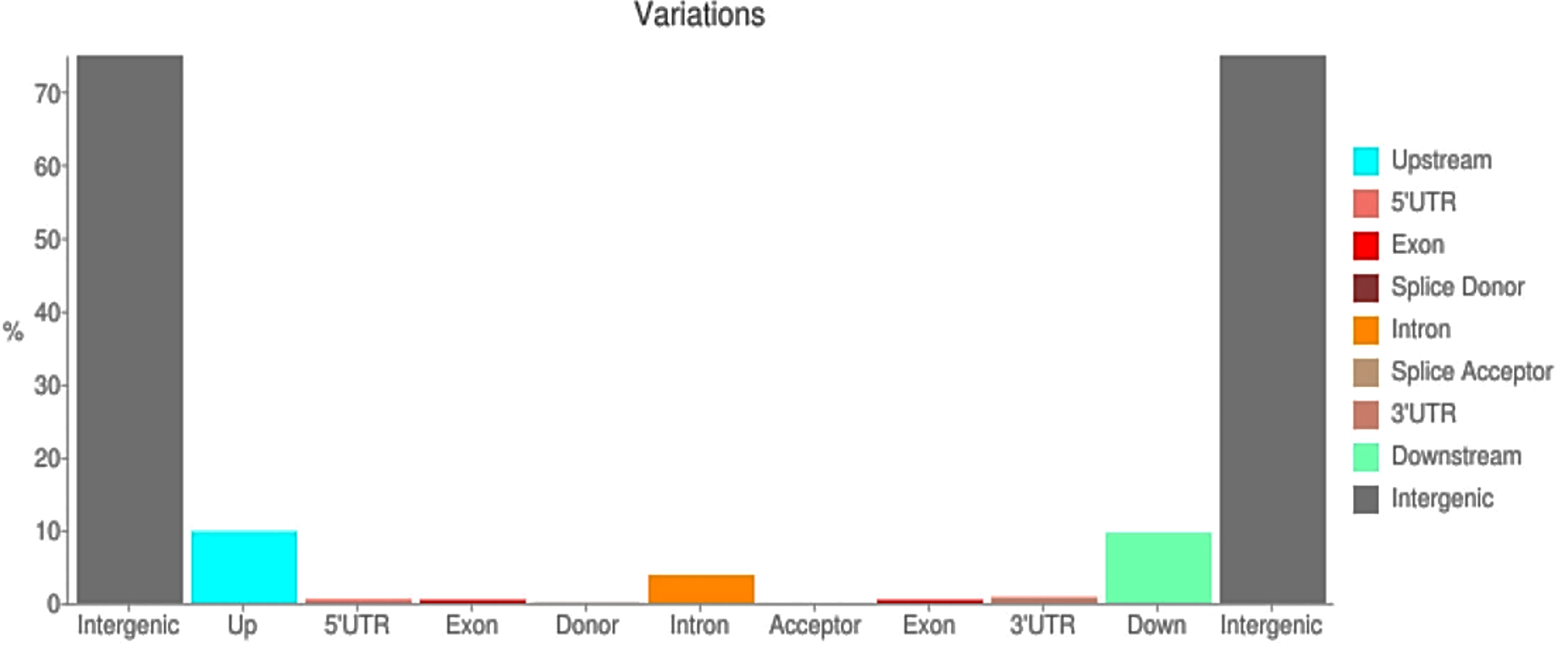

Supplement: S3 Fig — (TIF) [file pone.0302870.s003.tif]
