# Supplementary material for: Genome-wide discovery of InDels and validation of PCR-Based InDel markers for earliness in a RIL population and genotypes of lentil (Lens culinaris Medik.)
Source: PLoS One. 2024 May 22;19(5):e0302870. doi: 10.1371/journal.pone.0302870 (PMC11111061; doi:10.1371/journal.pone.0302870)
Supplement: S1 Table — (DOCX) [file pone.0302870.s004.docx]

**Table S1. Days to flowering (DTF) of RIL population and selected genotypes.**

| **RIL No.** | **DTF** |
| --- | --- |
| 1 | 63 |
| 2 | 60 |
| 3 | 56 |
| 4 | 88 |
| 5 | 57 |
| 6 | 60 |
| 7 | 63 |
| 8 | 88 |
| 9 | 60 |
| 10 | 57 |
| 11 | 56 |
| 12 | 60 |
| 13 | 84 |
| 14 | 60 |
| 15 | 60 |
| 16 | 84 |
| 17 | 88 |
| 18 | 56 |
| 19 | 91 |
| 20 | 60 |
| 21 | 60 |
| 22 | 91 |
| 23 | 91 |
| 24 | 60 |
| 25 | 84 |
| 26 | 60 |
| 27 | 60 |
| 28 | 57 |
| 29 | 84 |
| 30 | 57 |
| 31 | 91 |
| 32 | 91 |
| 33 | 55 |
| 34 | 60 |
| 35 | 57 |
| 36 | 88 |
| 37 | 88 |
| 38 | 68 |
| 39 | 56 |
| 40 | 88 |
| 41 | 62 |
| 42 | 88 |
| 43 | 57 |
| 44 | 62 |
| 45 | 57 |
| 46 | 57 |
| 47 | 91 |
| 48 | 73 |
| 49 | 55 |
| 50 | 91 |
| 51 | 91 |
| 52 | 55 |
| 53 | 93 |
| 54 | 91 |
| 55 | 60 |
| 56 | 96 |
| 57 | 91 |
| 58 | 56 |
| 59 | 57 |
| 60 | 56 |
| 61 | 91 |
| 62 | 60 |
| 63 | 96 |
| 64 | 55 |
| 65 | 60 |
| 66 | 91 |
| 67 | 91 |
| 68 | 57 |
| 69 | 60 |
| 70 | 60 |
| 71 | 60 |
| 72 | 57 |
| 73 | 63 |
| 74 | 60 |
| 75 | 98 |
| 76 | 60 |
| 77 | 60 |
| 78 | 60 |
| 79 | 89 |
| 80 | 56 |
| 81 | 57 |
| 82 | 55 |
| 83 | 55 |
| 84 | 60 |
| 85 | 60 |
| 86 | 56 |
| 87 | 60 |
| 88 | 56 |
| 89 | 60 |
| 90 | 60 |
| 91 | 57 |
| 92 | 56 |
| 93 | 55 |
| 94 | 60 |
| 95 | 57 |
| 96 | 57 |
| 97 | 60 |
| 98 | 56 |
| 99 | 57 |
| 100 | 57 |
| 101 | 63 |
| 102 | 57 |
| 103 | 84 |
| 104 | 56 |
| 105 | 57 |
| 106 | 89 |
| 107 | 60 |
| 108 | 84 |
| 109 | 84 |
| 110 | 91 |
| 111 | 89 |
| 112 | 56 |
| 113 | 56 |
| 114 | 57 |
| 115 | 91 |
| 116 | 63 |
| 117 | 91 |
| 118 | 70 |
| 119 | 56 |
| 120 | 55 |
| 121 | 57 |
| 122 | 55 |
| 123 | 56 |
| 124 | 60 |
| 125 | 60 |
| 126 | 58 |
| 127 | 58 |
| 128 | 55 |
| 129 | 56 |
| 130 | 60 |
| 131 | 58 |
| 132 | 58 |
| 133 | 56 |
| 134 | 60 |
| 135 | 56 |
| 136 | 63 |
| 137 | 63 |
| 138 | 55 |
| 139 | 60 |
| 140 | 57 |
| 141 | 57 |
| 142 | 63 |
| 143 | 60 |
| 144 | 60 |
| 145 | 60 |
| 146 | 57 |
| 147 | 60 |
| 148 | 63 |
| 149 | 60 |
| 150 | 56 |
| 151 | 60 |
| 152 | 60 |
| 153 | 93 |
| 154 | 56 |
| 155 | 57 |
| 156 | 56 |
| 157 | 60 |
| 158 | 60 |
| 159 | 89 |
| 160 | 60 |
| 161 | 57 |
| 162 | 60 |
| 163 | 65 |
| 164 | 60 |
| 165 | 57 |
| 166 | 96 |
| 167 | 63 |
| 168 | 60 |
| 169 | 84 |
| 170 | 63 |
| 171 | 93 |
| 172 | 60 |
| 173 | 60 |
| 174 | 94 |
| 175 | 100 |
| 176 | 63 |
| 177 | 60 |
| 178 | 57 |
| 179 | 55 |
| 180 | 58 |
| 181 | 57 |
| 182 | 84 |
| 183 | 63 |
| 184 | 56 |
| 185 | 60 |
| 186 | 60 |
| 187 | 84 |
| 188 | 89 |
| 189 | 60 |
| 190 | 57 |
| 191 | 60 |
| 192 | 90 |
| 193 | 89 |
| 194 | 90 |
| 195 | 84 |
| 196 | 60 |
| 197 | 60 |
| 198 | 56 |
| 199 | 56 |
| 200 | 57 |
| 201 | 89 |
| 202 | 57 |
| 203 | 60 |
| 204 | 60 |
| 205 | 56 |
| 206 | 56 |
| 207 | 57 |
| 208 | 86 |
| 209 | 63 |
| 210 | 91 |
| 211 | 60 |
| 212 | 60 |
| 213 | 84 |
| 214 | 60 |
| 215 | 57 |
| 216 | 56 |
| 217 | 60 |
| 218 | 60 |
| 219 | 57 |
| 220 | 57 |
| 221 | 57 |
| 222 | 63 |
| 223 | 57 |
| 224 | 60 |
| 225 | 60 |
| 226 | 60 |
| 227 | 60 |
| 228 | 57 |
| 229 | 59 |
| 230 | 56 |

| **Genotype** | **DTF** |
| --- | --- |
| L4775 | 58 |
| ILL7663 | 58 |
| Precoz | 63 |
| L4717 | 65 |
| L4727 | 70 |
| L830 | 80 |
| GM | 90 |
| MFX | 88 |
| L4602 | 85 |
