# Supplementary material for: Genome-wide discovery of InDels and validation of PCR-Based InDel markers for earliness in a RIL population and genotypes of lentil (Lens culinaris Medik.)
Source: PLoS One. 2024 May 22;19(5):e0302870. doi: 10.1371/journal.pone.0302870 (PMC11111061; doi:10.1371/journal.pone.0302870)
Supplement: S2 Table — (DOCX) [file pone.0302870.s005.docx]

**Table S2. Chromosome based InDel primers details in lentil.**

| **S. No.** | **Primer name** | **Forward primer** | **Reverse primer** | **Chrmosome** | **InDel position** | **InDels size** |
| --- | --- | --- | --- | --- | --- | --- |
|  | I-SP1-49.2 | CAGAAGGCATTGGGGACTAA | GCCTCATACGGTGCAATACC | Lcu.2RBY.Chr1 | 49226356 | 42 |
|  | I-SP1-110.4 | TGCTGTGAGTGGTGAAGAGG | ACCCTGCATGGTTTCTGTTC | Lcu.2RBY.Chr1 | 110399896 | 33 |
|  | I-SP1-121.9 | TGGCAAGCTAATTGCATCAC | TCAAACAATGCCCATTTTCA | Lcu.2RBY.Chr1 | 121887814 | 32 |
|  | I-SP1-251.2 | GGTCCTTGGATGACGTGAAG | GCCAGGCTCTCACAATTCTC | Lcu.2RBY.Chr1 | 251229599 | 33 |
|  | I-SP1-381.7 | TGATGACAACATGATGAATGCT | GGAGTAGGCGATGCAAAGAC | Lcu.2RBY.Chr1 | 381719188 | 38 |
|  | I-SP1-453.6 | TTCATTTTCTTCTTCGTTCCAAA | CACCTCCACAGTTTAATGGTGA | Lcu.2RBY.Chr1 | 453585869 | 49 |
|  | I-SP1-518.4 | GAAGAAGACGTTGGCTGGAG | ATGCAGGTTGAAAAGGGATG | Lcu.2RBY.Chr1 | 518431610 | 50 |
|  | I-SP2-40 | AAGTGCTTTTGCGTTCGACT | AAGCCAGCAAAGACCCCTAT | Lcu.2RBY.Chr2 | 40045827 | 34 |
|  | I-SP2-108.4 | TTTCCAAAGGAAGGGGAAAG | GAACAAATGGCTCCCGTAGA | Lcu.2RBY.Chr2 | 108401031 | 43 |
|  | I-SP2-219.4 | TTTTCCCCCAGCAAGTTTTA | GAAAAACGTACCGACCCAGA | Lcu.2RBY.Chr2 | 219356131 | 25 |
|  | I-SP2-327.8 | TTTTTGATCTGATAACTCTCTCTCTGA | CGTGACGCTCGATTTTATCA | Lcu.2RBY.Chr2 | 327823667 | 42 |
|  | I-SP2-401.4 | AGTCTTTGTTGCCTCCGAAA | TGGTTGTTGGTTGCTATGGA | Lcu.2RBY.Chr2 | 401439900 | 36 |
|  | I-SP2-534.3 | TCCCATCATGCTCATTTCAA | GAGGGAAAGTGGACACAACC | Lcu.2RBY.Chr2 | 534346530 | 45 |
|  | I-SP2-604.4 | GCAAAGAGAGCAATGCATGA | TCAACTTTTGCAGCATCAGG | Lcu.2RBY.Chr2 | 604405517 | 46 |
|  | I-SP3-93.7 | GAGCGCCCAAGATATATTCAA | AAGCCATCGGGAAGATAACC | Lcu.2RBY.Chr3 | 93743489 | 33 |
|  | I-SP3-153.8 | ACAAAACCCCTTGCTGAAGA | TCGATTTTGACATAGGAAGGAA | Lcu.2RBY.Chr3 | 153782210 | 29 |
|  | I-SP3-215.8 | CACTCAAAGCAACCGTGCTA | AACGAAAGCTTTGGATTGGA | Lcu.2RBY.Chr3 | 215775351 | 37 |
|  | I-SP3-328.8 | GGTCCCGTACGGATTTCATA | GGAGATGCTTACATCTGCACTT | Lcu.2RBY.Chr3 | 328785017 | 40 |
|  | I-SP-383.9 | AGCTAAGGCGCAAGTCCAAA | TGGGTGTGTTCAACCTAGTCG | Lcu.2RBY.Chr3 | 383964202 | 34 |
|  | I-SP3-402.9 | GGGTCGTACAAATGCGAGAT | TGATGTTACCACATGCACGA | Lcu.2RBY.Chr3 | 402853868 | 31 |
|  | I-SP4-6.1 | TGTGATGTGGTGATTGTTGG | AATCTCACCGATCCCATCTG | Lcu.2RBY.Chr4 | 6136828 | 30 |
|  | I-SP4-106.2 | TTGGTCCAGTTTTGGTTTACG | AGTTGAAGCAACGTGTGACG | Lcu.2RBY.Chr4 | 106248582 | 43 |
|  | I-SP4-266 | GCTGGGGATTTGTACAGGAA | CAGCGGAGACCTCAGAAAGT | Lcu.2RBY.Chr4 | 266032381 | 44 |
|  | I-SP4-310.6 | CAACAGAGGAATTTGATGTGG | TGGCCTCAATTTTCGTCATT | Lcu.2RBY.Chr4 | 310570037 | 30 |
|  | I-SP4-414.4 | ATATGAACCCGCAAGCTCAC | CAATAAACCCTGGCCTCAAA | Lcu.2RBY.Chr4 | 414351635 | 41 |
|  | I-SP5-41.1 | TGACTTGAAATCCACTAGCATAGG | GACACCACAAACCCTCACAG | Lcu.2RBY.Chr5 | 41096854 | 43 |
|  | I-SP5-235.5 | TTGGGGTACAATCTGGGAAG | CAACGGTTCACAAGTGGATG | Lcu.2RBY.Chr5 | 235464794 | 31 |
|  | I-SP5-311.8 | TCGGGTTTGAAAGAGGACTG | CCAATAACCTGCTCTGATACCAA | Lcu.2RBY.Chr5 | 311751064 | 46 |
|  | I-SP5-414.5 | GAAGCATGCTCAGCAATCAG | GCAATGCACATGCAAGTTTT | Lcu.2RBY.Chr5 | 414528734 | 30 |
|  | I-SP5-468.6 | CTTGACCTTTATTTAAACACTTGCT | TCGGTGTCTCCAAATTCTTTT | Lcu.2RBY.Chr5 | 468591471 | 39 |
|  | I-SP6-33.5 | AGGGGTGAGTTTCATTCCAA | TATCGGTACCGCATGCATAA | Lcu.2RBY.Chr6 | 33462995 | 37 |
|  | I-SP6-153.7 | ATTGAATCACAGGCCCAATC | CGTATGATGCACCTGGACCT | Lcu.2RBY.Chr6 | 153675384 | 37 |
|  | I-SP6-306.6 | TCGATTTCCCTTTTGTGAAATTA | GTTTGACATTTTTATGTCATTTGATT | Lcu.2RBY.Chr6 | 306602865 | 33 |
|  | I-SP6-404.3 | TGCTTTTTAGGGTTCGACCA | GGAGGTGCACGAGATTAGGA | Lcu.2RBY.Chr6 | 404285153 | 37 |
|  | I-SP7-111.7 | AGGCACACAGCACATGAGAG | CCGGGAATTCCTAAAGGCTA | Lcu.2RBY.Chr7 | 111652362 | 40 |
|  | I-SP7-205.6 | GCATTGTCAGCCTCATCAAA | GCAGATGCTTTTGGCTGAAT | Lcu.2RBY.Chr7 | 205574902 | 34 |
|  | I-SP7-426.1 | CACATCAGCACATCCACACA | TGCATGCATTGAGTCAGTTG | Lcu.2RBY.Chr7 | 426078789 | 26 |
