# Supplementary material for: Genome-wide discovery of InDels and validation of PCR-Based InDel markers for earliness in a RIL population and genotypes of lentil (Lens culinaris Medik.)
Source: PLoS One. 2024 May 22;19(5):e0302870. doi: 10.1371/journal.pone.0302870 (PMC11111061; doi:10.1371/journal.pone.0302870)
Supplement: S1 Raw images — (PDF) [file pone.0302870.s006.pdf]

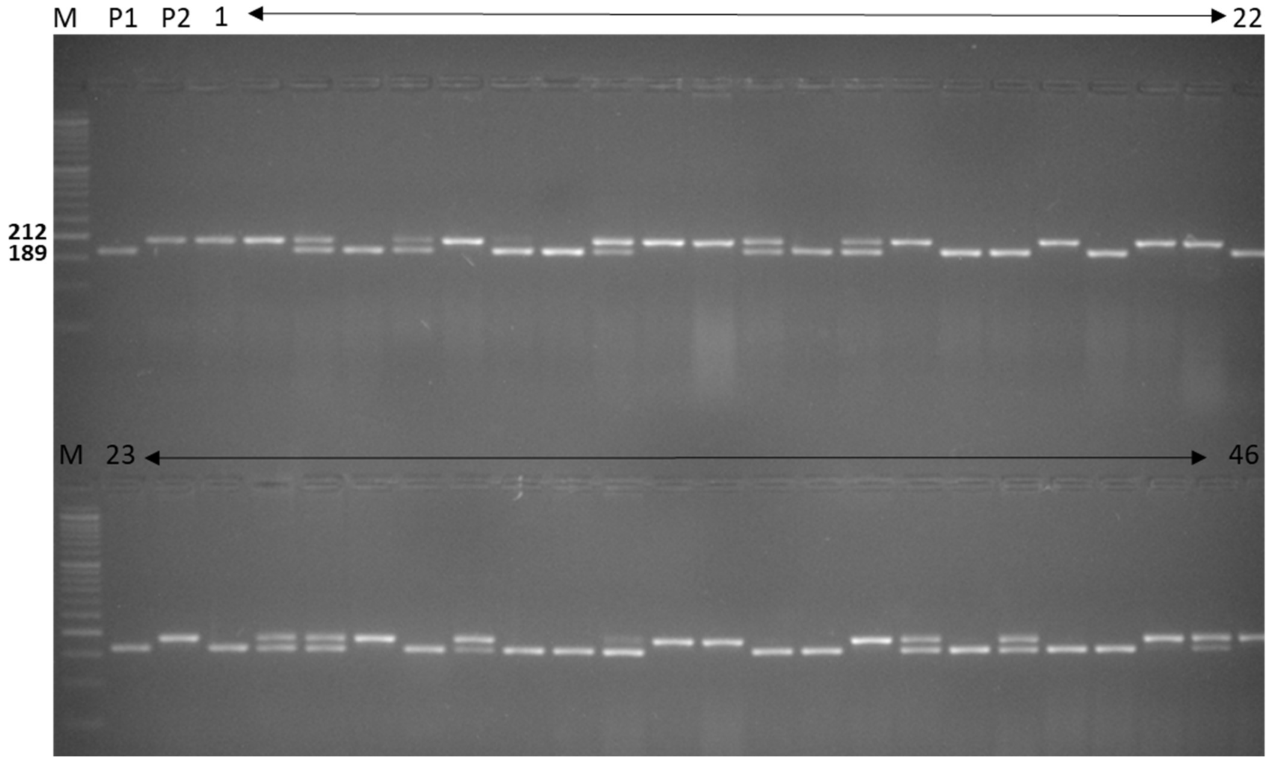

(a)

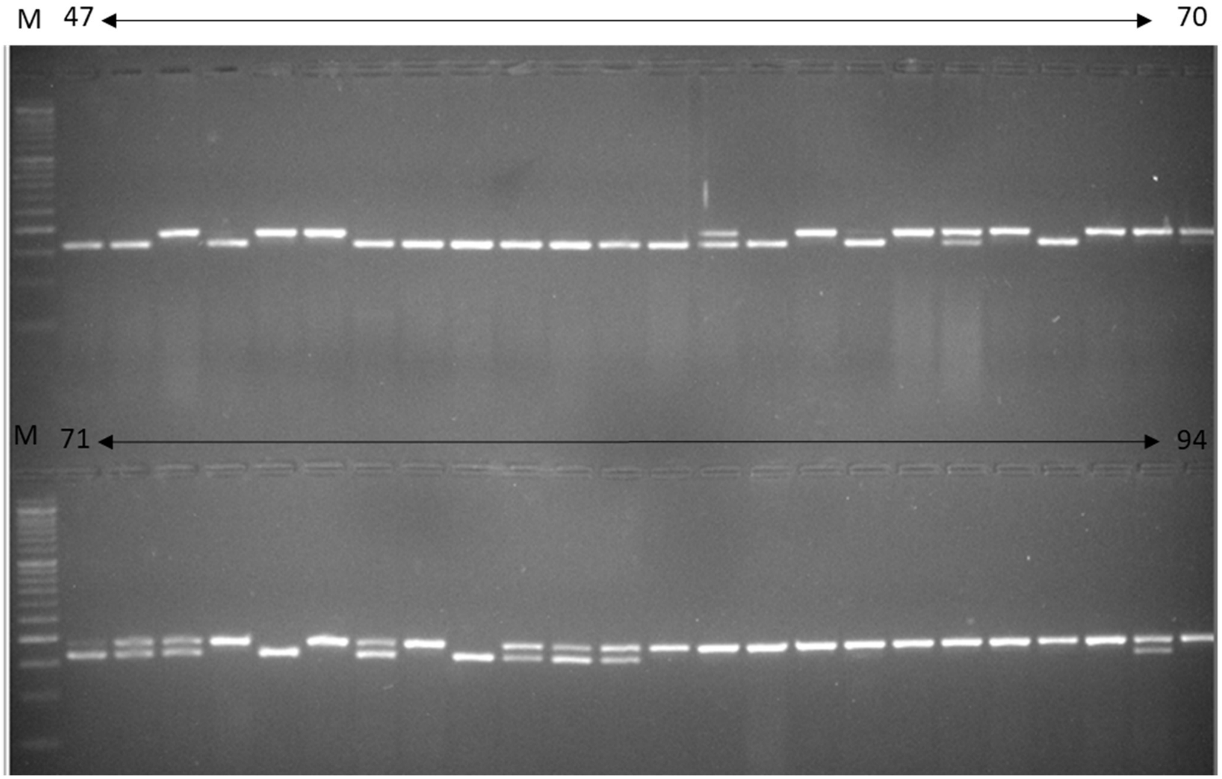

(b)

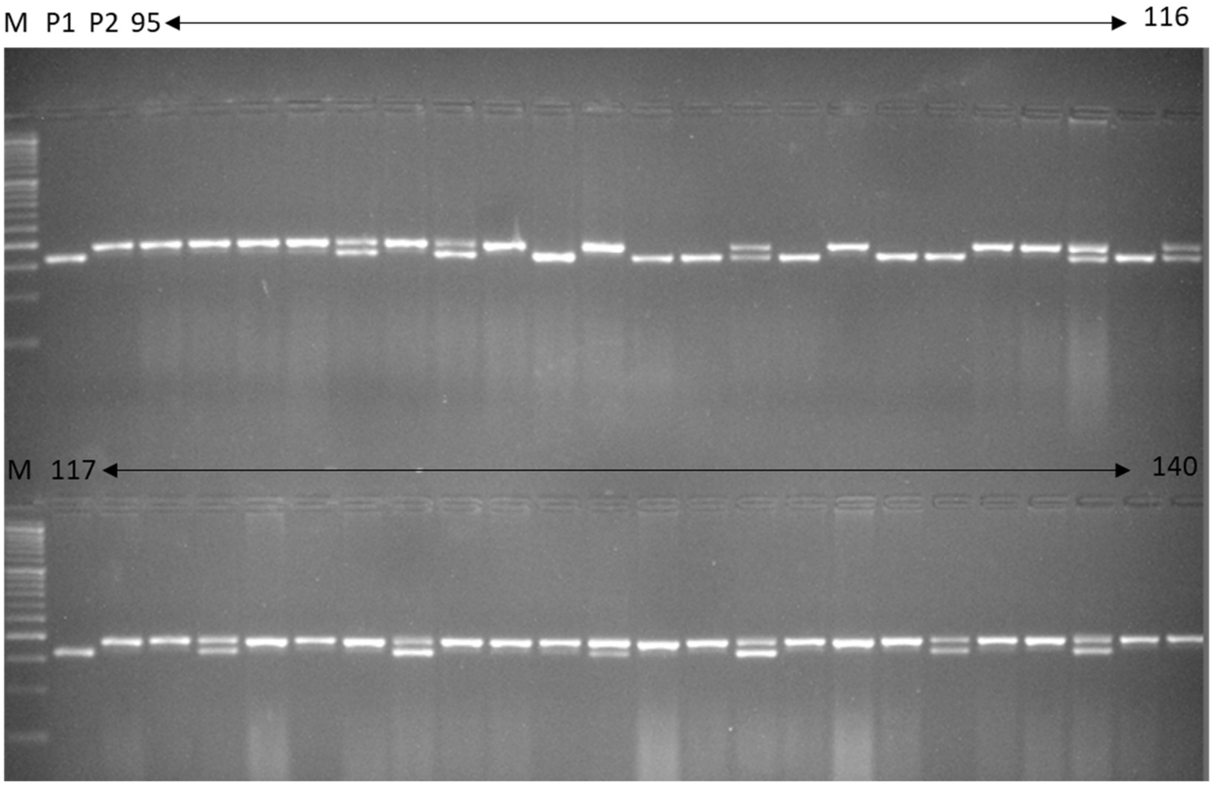

(c)

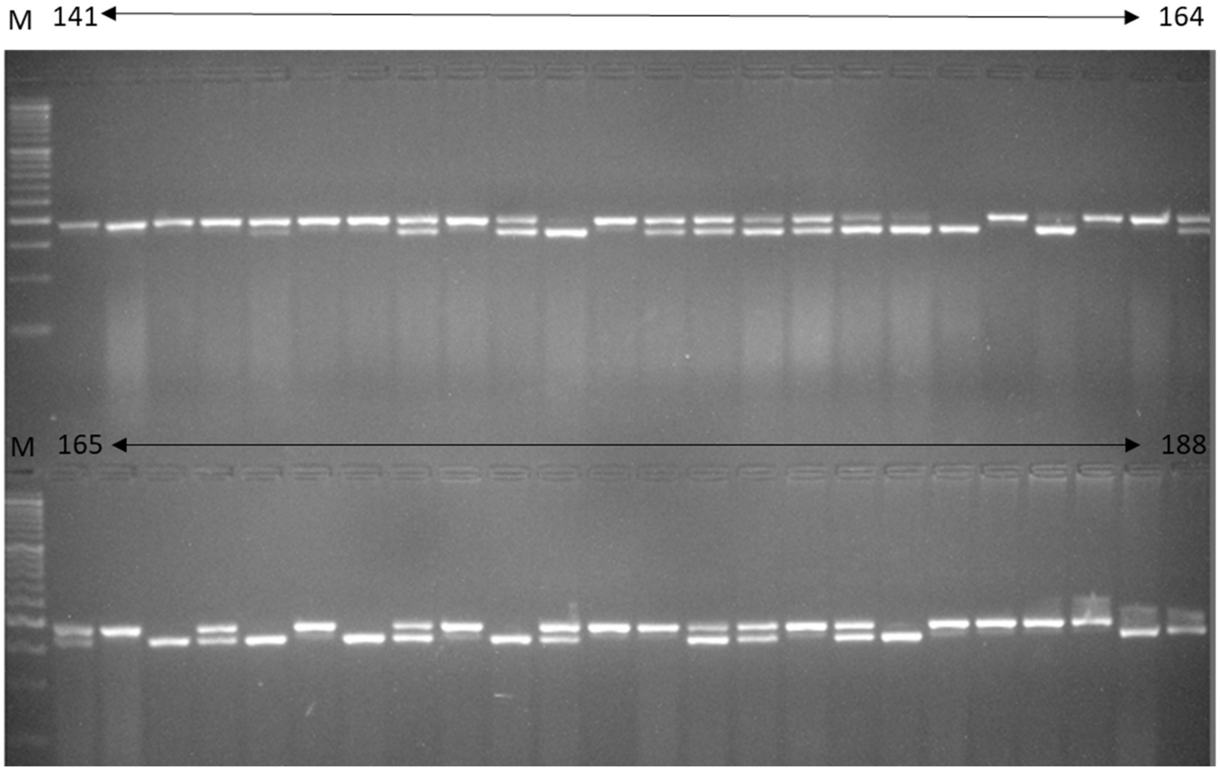

(d)

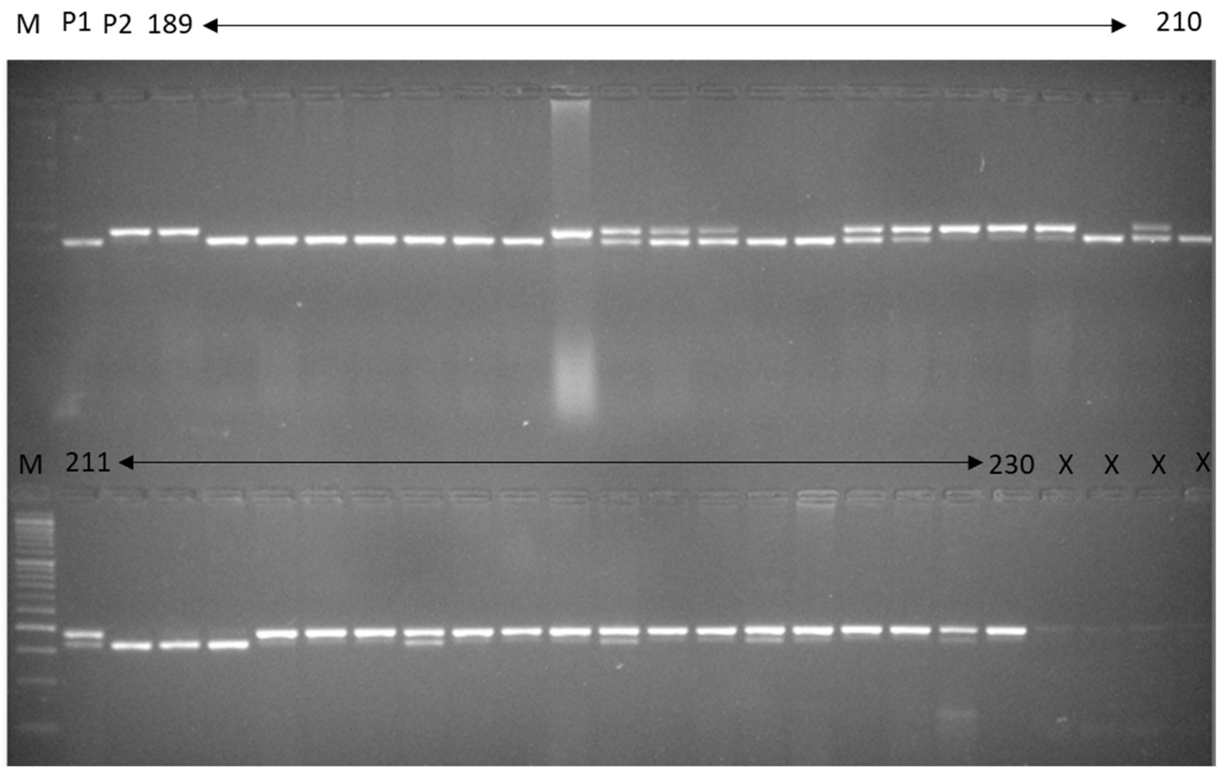

(e)

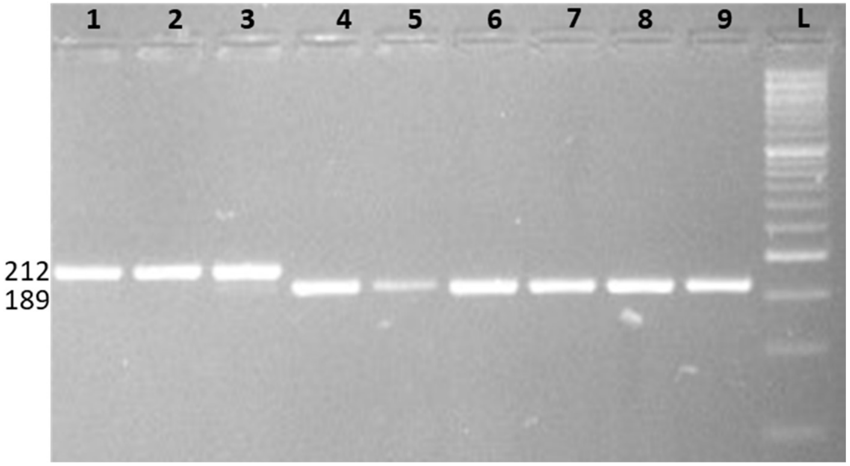

(f)

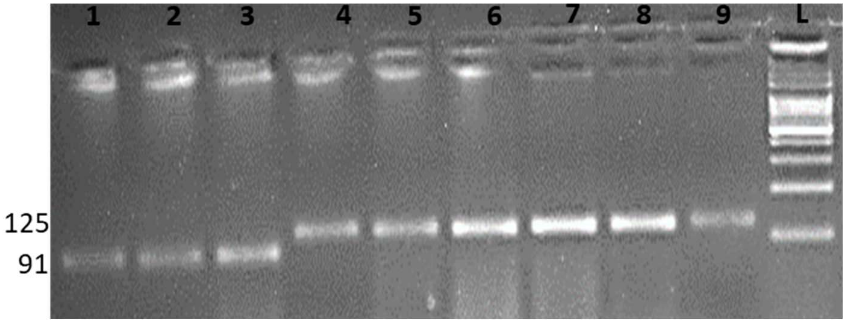

(g)

Fig. S4. (a-e). Raw images of genotyping of 230 RIL individuals using InDel marker I-SP-356.6; (f-g) Raw images of InDel marker polymorphism details in nine lentil genotypes differing for maturity duration. Where, 1. L4775; 2. ILL7663; 3. Precoz; 4. L830; 5. L4717; 6. L4727; 7. Globe mutant; 8. MFX; 9. L4602
